# Dataset used in the economic evaluation trastuzumab-based regimens for HER-2 positive metastatic breast cancer patients in the Taiwanese healthcare setting

**DOI:** 10.1016/j.dib.2020.105194

**Published:** 2020-01-25

**Authors:** Vakaramoko Diaby, Ching-Yu Wang, Hussain Alqhtani, Sascha van Boemmel-Wegmann, Askal Ayalew Ali, Rajesh Balkrishnan, Yu Ko, Sofia Palacio, Gilberto de Lima Lopes

**Affiliations:** a Department of Pharmaceutical Outcomes and Policy, College of Pharmacy, University of Florida, Gainesville, Florida, USA; b Department of Clinical Pharmacy, College of Pharmacy, Najran University, Najran, Kingdom of Saudi Arabia; c Economic, Social & Administrative Pharmacy, College of Pharmacy and Pharmaceutical Sciences, Florida A&M University, Tallahassee, Florida, USA; d Population Health and Prevention Research, University of Virginia School of Medicine, Charlottesville, Virginia, USA; e School of Pharmacy, College of Pharmacy, Taipei Medical University, Taipei, Taiwan; f Sylvester Comprehensive Cancer Center at the University of Miami and the Miller School of Medicine, Miami, Florida, USA

**Keywords:** Markov model, Metastatic breast cancer, Cost-effectiveness analysis, Treatment sequence

## Abstract

The present data article aims to describe the input parameters for a Markov model assessing the cost-effectiveness of four treatment sequences for patients with HER-2 positive metastatic breast cancer. The model input parameters include costs for physician visits, drugs, adverse event management, computed tomography (CT) scan, laboratory tests, echocardiogram, utilities, disutilities as well as the shape and scale parameters of a log-logistic distribution used for the transition probabilities.

**Table:** Specifications Table

Subject	Economics and Econometrics
Specific subject area	Cost-effectiveness analysis
Type of data	Tables
How data were acquired	Data were obtained from clinical trials, published literature and Taiwanese National Health Insurance Administration's website.
Data format	Raw and analyzed data
Parameters for data collection	Data were obtained for metastatic breast cancer patients in the Taiwanese setting.
Description of data collection	Cost data were mostly obtained from the Taiwanese National Health Insurance Administration website and further calculated based on average weight and body surface area for Taiwanese females. Transition probabilities were estimated following a survival analysis of approximated individual patient data from published Kaplan-Meier survival curves of clinical trials. Utilities were obtained from published literature.
Data source location	Taiwan and literature
Data accessibility	Data were included in this article
Related research article	A cost-effectiveness analysis of Trastuzumab-Containing Treatment Sequences for HER-2 Positive Metastatic Breast Cancer Patients in Taiwan. *The Breast (Revision submitted).*

**Table:** 

**Value of the Data** • Our data provide input parameters for cost, utilities and transition probabilities for cost-effectiveness analysis (CEA) of Trastuzumab-based regimens for metastatic breast cancer patients in Taiwan. • Researchers conducting economic evaluation including cost-effectiveness analysis and cost of illness studies for metastatic breast cancer patients would benefit from these data. • Parenteral drug costs provided in this article are adjusted for wastage through a scenario analysis.

## 1 Data

The dataset includes the model input parameters for a Markov model assessing the cost-effectiveness of four trastuzumab containing sequences for HER-2 positive metastatic breast cancer patients as well as a brief description of the model assumptions. These input parameters include cost, transition probabilities, and utilities. Cost data include physician visit fee (Table 1), treatment acquisition cost both considering or not considering drug wastage (Table 2), costs associated with adverse event management (Table 2, Table 3), costs for computed tomography (CT) scan and echocardiogram and laboratory costs (Table 4). For transition probabilities, the shape and scale parameters for progression-free survival and overall survival were estimated as well as the probability of developing adverse events while on different treatment regimens. The entire list of model input parameters including the lower and higher bounds for deterministic sensitivity analysis as well as the distribution and standard deviations for probabilistic sensitivity analyses are shown in Table 5, Table 6 for base case and no drug wastage scenarios respectively.

**Table 1:** Physician visit costs and treatment acquisition costs.

Input parameters - Costs	Assumption and cost calculation
Monthly cost for physician visit	▪ Cost for physician visit = $8.15 [1] ▪ Visit every 3 weeks or every 0.69 months ▪ 8.15*0.69 = 4.348125
Base case scenario	
Acquisition cost of treatments— Pertuzumab, Docetaxel, Trastuzumab [PTH] vs. Docetaxel/Trastuzumab [TH] [2]	▪ Assumption: Patients received a maximum of 6 cycles of docetaxel. Trastuzumab/pertuzumab alone was continued to avoid neuropathy in case patients responded to treatments. ▪ Drug Costs (per unit) were Average Sale Prices (ASP) from National Health Insurance Administration Ministry of Health and Welfare (March 2017) [3,4] ▪ Cost of docetaxel: 40mg/ml 0.5ml vial, $132.68; 40mg/ml 2ml vial, $500.50; 75 mg/m2, assuming body surface area (BSA) of 1.59 = 119.25 mg = 1 vial 40mg/ml 2ml + 2x 40mg/ml 0.5ml = $765.86 ▪ Cost of Trastuzumab: 440 mg vial, $1852.07; Day 1 Cycle 1 is 8 mg/kg, which would be 464.8 mg = 2 × 440mg = $3704.14 for an average person weighing 58.1 Kg; Subsequent cycles 6 mg/kg which would be 348.6 mg = 1 × 440 mg = $1852.07 for an average person weighing 58.1 Kg ▪ Cost of pertuzumab: 420 mg, $2282.21; Initial cycle 1 dose is 840 mg, or $4564.42; Subsequent doses are 420 mg or $2282.21 ▪ Cost of Pegfilgrastim: 6mg, $662.68 per cycle cost for 6 cycles ▪ **Total weekly cost of Pertuzumab, Trastuzumab, Docetaxel [THP], plus pegfilgrastim**: week 1: $9697.10 = $765.86+$3704.14+$4564.42+$662.68 weeks 4,7,10,13,16: $5562.82 = $765.86+$1852.07+$2282.21+$662.68 weeks 17,20,23,26, 29, and beyond (no docetaxel): $4134.28 = $1852.07+$2282.21 ▪ **Total weekly cost of Trastuzumab, Docetaxel [TH], no pegfilgrastim:** week 1: $4470.00 = $765.86+$3704.14 weeks 4,7,10,13,16: $2617.93 = $765.86+$1852.07 weeks 17,20,23,26, 29, and beyond (no docetaxel): $1852.07
Acquisition cost of treatments— Trastuzumab emtansine (TDM-1)	▪ Assumption: patient received TDM-1 every 3 weeks ▪ Drug Costs (per unit) were Average Sale Prices (ASP) from National Health Insurance Administration Ministry of Health and Welfare (March 2017) [3,4] ▪ Cost of TDM-1: 10 mg, $1794.71; 160 mg, $2871.77; dose is 3.6 mg/kg which is 209.16 mg for an average person weighing 58.1 Kg; 1 × 160mg + 1 × 100 mg; $4666.48 every 21 days ▪ **Total cost of TDM-1 (every 3 weeks): $4666.48**
Acquisition cost of treatments— Lapatinib + Capecitabine (Xeloda)	▪ Drug Costs (per unit) were Average Sale Prices (ASP) from National Health Insurance Administration Ministry of Health and Welfare (March 2017) [3,4] ▪ Cost of Lapatinib: 1500 mg, $97.74 (daily); weekly cost of lapatinib is $684.18 ▪ Cost of Capecitabine: 500 mg, $3.23; dose is 2000 mg/m2 daily which is 3180 mg or 7 tablets daily for an average person with BSA of 1.59; $22.61 per day; x14 days on with 1 week rest; weekly cost of capecitabine is $158.27 ($22.61x7), assuming 2 weeks of treatment per cycle ▪ **Total cost of Lapatinib + Capecitabine:** Weeks 1 and 2, weekly cost is: $842.45=($684.18 + 158.27) Week 3 weekly cost is: $684.18 Week 4, weekly cost is: $842.45=($684.18 + 158.27)
Acquisition cost of treatments— Trastuzumab + Lapatinib	▪ Drug Costs (per unit) were Average Sale Prices (ASP) from National Health Insurance Administration Ministry of Health and Welfare (March 2017) [3,4] ▪ Cost of Trastuzumab: Loading Dose trastuzumab 8 mg/kg which is 464.8 mg for an average person weighing 58.1kg; 2 × 440mg; $3704.14; Maintenance dose trastuzumab 6 mg/kg which is 348.6 mg for an average person weighing 58.1kg; 1 × 440 mg every 3 weeks; $1852.07 ▪ Cost of Lapatinib: 1500 mg, $97.74 (daily); weekly cost of lapatinib is $684.18 ▪ **Total cost of Trastuzumab + Lapatinib:** Week 1: $4388.32 = $3704.14+$684.18 Week 2,3: $684.18 Week 4: $2536.25 = $1852.07+$684.18 Week 5,6: $684.18 Week 7,10,13,16, etc: $2536.25 Week 8,9,11,12,14,15,17,18, etc.: $684.18
Acquisition cost of treatments—Trastuzumab + Capecitabine	▪ Drug Costs (per unit) were Average Sale Prices (ASP) from National Health Insurance Administration Ministry of Health and Welfare (March 2017) [3,4] ▪ Cost of Trastuzumab: Loading Dose trastuzumab 8 mg/kg which is 464.8 mg for an average person weighing 58.1kg; 2 × 440mg; $3704.14; Maintenance dose trastuzumab 6 mg/kg which is 348.6 mg for an average person weighing 58.1kg; 1 × 440 mg every 3 weeks; $1852.07 ▪ Cost of Capecitabine: 500 mg, $3.23; dose is 2000 mg/m2 daily which is 3180 mg or 7 tablets daily for an average person with BSA of 1.59; $22.61 per day; x14 days on with 1 week rest; weekly cost of capecitabine is $158.27 ($22.61x7), assuming 2 weeks of treatment per cycle ▪ **Total cost of Trastuzumab + Capecitabine:** Week 1: $3862.41 = $3704.14+$158.27 Week 2: $158.27 Week 3: $3704.14 Week 4: $2010.34 = $1852.07+$158.27 Week 5: $158.27 Week 6: $3704.14 Week 7: $2010.34 = $1852.07+$158.27 Week 8: $158.27 Week 9: $3704.14 Weeks 10, 13, 16, 19, 22, etc…: $2010.34 = $1852.07+$158.27 Weeks 11,14,17,20,23, etc…:$158.27 Weeks 12,15,18,21,24, etc…:$3704.14
No drug wastage scenario	
Acquisition cost of treatments— Pertuzumab, Docetaxel, Trastuzumab [PTH] vs. Docetaxel/Trastuzumab [TH] [2]	▪ Assumption: Patients received a maximum of 6 cycles of docetaxel, and then if they were responding trastuzumab/pertuzumab alone was continued to avoid neuropathy. ▪ Drug Costs (per unit) were Average Sale Prices (ASP) from National Health Insurance Administration Ministry of Health and Welfare (March 2017) [3,4] ▪ Cost of docetaxel: 40mg/ml 0.5ml vial, $132.68; 40mg/ml 2ml vial, $500.50; 75 mg/m2, assuming body surface area (BSA) of 1.59 = 119.25 mg = 1.49 vial 40mg/ml 2ml = $760.88 ▪ Cost of trastuzumab: 440 mg vial, $1852.07; Day 1 Cycle 1 is 8 mg/kg, which would be 464.8 mg = 1.056 vials = $1955.79 for an average person weighing 58.1 Kg; Subsequent cycles 6 mg/kg which would be 348.6 mg = 0.792 vials = $1466.84 for an average person weighing 58.1 Kg ▪ Cost of pertuzumab: 420 mg, $2282.21; Initial cycle 1 dose is 840 mg, or $4564.42; Subsequent doses are 420 mg or $2282.21 ▪ Cost of Pegfilgrastim: 6mg, $662.68 per cycle cost for 6 cycles ▪ **Total weekly cost of Pertuzumab, Trastuzumab, Docetaxel [THP], plus pegfilgrastim:** week 1: $7,943,77 = $760.88+$1955.79+$4564.42+$662.68 weeks 4,7,10,13,16: $5172.61 = $760.88+$1466.84+$2282.21+$662.68 weeks 17,20,23,26, 29, and beyond (no docetaxel): $3749.05 = $1466.84+$2282.21 ▪ **Total weekly cost of Trastuzumab, Docetaxel [TH], no pegfilgrastim:** week 1: $2716.67 = $760.88+$1955.79 weeks 4,7,10,13,16: $2227.72 = $760.88+$1466.84 weeks 17,20,23,26, 29, and beyond (no docetaxel): $1466.84
Acquisition cost of treatments—Trastuzumab emtansine (TDM-1)	▪ Drug Costs (per unit) were Average Sale Prices (ASP) from National Health Insurance Administration Ministry of Health and Welfare (March 2017) [3,4] ▪ Assumption: patient received TDM-1 every 3 weeks ▪ Cost of TDM-1: 10 mg, $1794.71; 160 mg, $2871.77; dose is 3.6 mg/kg which is 209.16 mg for an average person weighing 58.1 Kg; 1 × 160mg + 0.4915 × 100mg; $3754.05 every 21 days ▪ **Total cost of TDM-1 (every 3 weeks): $3754.05**
Acquisition cost of treatments— Lapatinib + Capecitabine (Xeloda)	▪ Drug Costs (per unit) were Average Sale Prices (ASP) from National Health Insurance Administration Ministry of Health and Welfare (March 2017) [3,4] ▪ Cost of Lapatinib: 1500 mg, $97.74 (daily); weekly cost of lapatinib is $684.18 ▪ Cost of Capecitabine: 500 mg, $3.23; dose is 2000 mg/m2 daily which is 3180 mg or 7 tablets daily for an average person with BSA of 1.59; $22.61 per day; x14 days on with 1 week rest; weekly cost of capecitabine is $158.27 ($22.61x7), assuming 2 weeks of treatment per cycle ▪ **Total cost of Lapatinib + Capecitabine:** Weeks 1 and 2, weekly cost is: $842.45=($684.18 + 158.27) Week 3 weekly cost is: $684.18< Week 4, weekly cost is: $842.45=($684.18 + 158.27)
Acquisition cost of treatments— Trastuzumab + Lapatinib	▪ Drug Costs (per unit) were Average Sale Prices (ASP) from National Health Insurance Administration Ministry of Health and Welfare (March 2017) [3,4] ▪ Cost of Trastuzumab: Loading Dose trastuzumab 8 mg/kg which is 464.8 mg for an average person weighing 58.1kg; 1.056 × 440mg; $1955.79; Maintenance dose trastuzumab 6 mg/kg which is 348.6 mg for an average person weighing 58.1kg; 0.792 × 440 mg every 3 weeks; $1466.84 ▪ Cost of Lapatinib: 1500 mg, $97.74 (daily); weekly cost of lapatinib is $684.18 ▪ Total cost of Trastuzumab + Lapatinib: Week 1: $2639.97 = $1955.79+$684.18 Week 2,3: $684.18 Week 4: $2151.02 = $1466.84+$684.18 Week 5,6: $684.18 Week 7,10,13,16, etc: $2151.02 Week 8,9,11,12,14,15,17,18, etc.: $684.18
Acquisition cost of treatments—Trastuzumab + Capecitabine	▪ Drug Costs (per unit) were Average Sale Prices (ASP) from National Health Insurance Administration Ministry of Health and Welfare (March 2017) [3,4] ▪ Cost of Trastuzumab: Loading Dose trastuzumab 8 mg/kg which is 464.8 mg for an average person weighing 58.1kg; 1.056 × 440mg; $1955.79 ▪ Maintenance dose trastuzumab 6 mg/kg which is 348.6 mg for an average person weighing 58.1kg; 0.792 × 440 mg every 3 weeks; $1466.84 ▪ Cost of Capecitabine: 500 mg, $3.23; dose is 2000 mg/m2 daily which is 3180 mg or 7 tablets daily for an average person with BSA of 1.59; $22.61 per day; x14 days on with 1 week rest; weekly cost of capecitabine is $158.27 ($22.61x7), assuming 2 weeks of treatment per cycle ▪ **Total cost of Trastuzumab + Capecitabine:** Week 1: $2114.06 = $1955.79+$158.27 Week 2: $158.27 Week 3: $1955.79 Week 4: $1625.11 = $1466.84+$158.27< Week 5: $158.27 Week 6: $1955.79 Week 7: $1625.11 = $1466.84+$158.27< Week 8: $158.27 Week 9: $1955.79 Weeks 10, 13, 16, 19, 22, etc…: $1625.11 = $1466.84+$158.27 Weeks 11,14,17,20,23, etc…:$158.27 Weeks 12,15,18,21,24, etc…:$1955.79

**Table 2:** Cost associated with adverse event management.

Adverse events	Costs in 2018 USD			Reference
	Average	Lower bound	Upper bound	
Diarrhea	3586.31	1766.39	7172.62	Niraula et al. [5]
Neutropenia	6904.98	3532.78	18038.6	Niraula et al. [5]
Febrile neutropenia	22481.34	11240.67	46033.23	Niraula et al. [5]
Thrombocytopenia	18199.18	9046.06	35863.1	Niraula et al. [5]
Hand-foot syndrome/Palmar–plantar erythrodysesthesia/skin changes	2034.03	1017.01	4121.58	Niraula et al. [5]
Rash	321.16	160.58	535.27	Niraula et al. [5]
Nausea/Vomiting	6958.51	3479.26	13917.02	Niraula et al. [5]
Fatigue	1017.01	535.27	909.96	Niraula et al. [5]
Dyspnea	4720.63	–	–	Sharpe [6]
Cardiovascular disorder	2410.24	1826.86	4871.63	Garrison et al. [7]

*Original costs were inflated to represent 2018 U.S. dollars costs using the Consumer Price Index (CPI) inflation calculator from the Bureau Labor of Statistics (available at http://www.bls.gov/data/inflation_calculator.htm)

**Table 3:** Estimated per-patient cost (2018 US dollars)^a^of managing treatment-related grade 3 and above adverse events that occurred in ≥ 5% of patients.

Adverse event	Pertuzumab + Trastuzumab + Docetaxel	Trastuzumab + Docetaxel	TDM1	Lapatinib + Capecitabine	Trastuzumab + Lapatinib	Trastuzumab + Capecitabine
Trial	Swain et al. [2]	Swain et al. [2]	Verma et al. [8]	Geyer et al. [9]	Blackwell et al. [10]	von Minckwitz et al. [11]
Diarrhea	322.76 (158.97; 645.53)	–	–	459.05 (226.10; 918.10)	251.05 (123.65; 502.09)	191.15 (94.15; 382.30)
Neutropenia	3383.44 (1731.06; 8838.92)	3176.29 (1625.08; 8297.75)	–	–	–	–
Febrile neutropenia	2922.58 (1461.29; 5984.33)	1573.69 (786.85; 3222.32)	–	–	–	–
Thrombocytopenia	–	–	2347.69 (1166.94; 4626.34)	–	–	–
Hand-foot syndrome/Palmar–plantar erythrodysesthesia/skin changes	–	–	–	142.38 (71.19; 288.50)	–	660.44 (330.22; 1338.27)
Rash	–	–	–	–	70.65 (35.32; 117.76)	–
Nausea/Vomiting	–	–	–	–	–	–
Fatigue	–	–	–	–	–	–
Dyspnea	–	–	–	–	–	–
Cardiovascular disorder	–	–	–	–	–	125.09 (94.82; 252.83)
Total	6628.78 (3351.33; 115468.77)	4749.98 (2411.92; 11520.08)	2347.69 (1166.94; 4626.34)	601.43 (297.29: 1206.61)	321.70 (158.98; 619.85)	976.68 (519.18; 1973.40)

a Original costs were inflated to represent 2018 U.S. dollars costs using the Consumer Price Index (CPI) inflation calculator from the Bureau Labor of Statistics (available at http://www.bls.gov/data/inflation_calculator.htm); Anorexia and headache were excluded based on clinical expert opinion; -: Adverse events occurred in less than 5% of patients.

**Table 4:** Costs and assumptions for computed tomography (CT) scan, laboratory tests, echocardiogram.

Cost input parameter	Assumption and cost calculation
Computed tomography (CT) scan [1]	▪ Assumption: CT scan every 2 months or 8.69625 weeks ▪ Reimbursement item code of National Health Insurance: 33071B CT thorax/abdomen/pelvis ($142.86) ▪ **Cost of CT scan (every 9 weeks):** $142.86
Laboratory tests [1]	▪ Assumption: Every three weeks or 0.69 months ▪ Comprehensive metabolic panel ($15.71); Complete blood count ($6.27) ▪ **Cost of blood tests (every three weeks):** $22.98
Echocardiogram [1]	▪ Assumption: Only for patients receiving HER2 therapy, every 3 months or 13.044 weeks ▪ Echocardiography, transthoracic, real-time with image documentation ($99.52) ▪ **Cost of an echocardiogram (every 13 weeks):** $99.52

**Table 5:** Model input parameters for base case scenario.

Parameters	Unit	Baseline	Deterministic SA		Probabilistic SA		Assumptions
			Low	High	PSA (SD)	Distribution	
Medical visit							
Physician fees	Every 3 weeks	8.15	6.11	10.19	1.01875	Gamma	25% +/− rule
Acquisition cost of treatments							
Loading dose pertuzumab (840 mg)	1st week of treatment only	4564.42	2282.21	6846.63	1141.105	Gamma	50% +/− rule
Maintenance dose pertuzumab (420 mg)	Every 3 weeks starting from week 4	2282.21	1141.11	3423.32	570.5525	Gamma	50% +/− rule
Loading dose trastuzumab (8mg/kg) -	1st week of treatment only	3704.14	1852.07	5556.21	926.035	Gamma	50% +/− rule
Maintenance dose trastuzumab (6 mg/kg)	Every 3 weeks starting from week 4	1852.07	926.04	2778.11	463.0175	Gamma	50% +/− rule
Docetaxel (1 mg)	Every 3 weeks for 6 cycles	765.86	382.93	1148.79	191.465	Gamma	50% +/− rule
Pegfilgrastim (6 mg)	Every 3 weeks for 6 cycles	662.68	331.34	994.02	165.67	Gamma	50% +/− rule
TDM1	Every 3 weeks	4666.48	2333.24	6999.72	1166.62	Gamma	50% +/− rule
Capecitabine (500 mg)	Assuming 2 weeks of treatment per cycle + 1 week rest	158.27	79.14	237.41	39.5675	Gamma	50% +/− rule
Lapatinib (1500 mg daily)	Weekly	684.18	342.09	1026.27	171.045	Gamma	50% +/− rule
Cost of the management of Adverse Events (grade 3/4)							
Pertuzumab + trastuzumab + docetaxel	1 time	6628.784	3351.325	15468.774	3029.362	Gamma	Calculated
Trastuzumab + docetaxel	1 time	4749.982	2411.924	11520.075	2277.038	Gamma	Calculated
T-DM1	1 time	2347.695	1166.942	4626.3412	864.8498	Gamma	Calculated
Lapatinib + capecitabine	1 time	601.4326	297.29	1160.6912	215.8503	Gamma	Calculated
Trastuzumab + lapatinib	1 time	321.70	158.9764	619.84961	115.2183	Gamma	Calculated
Trastuzumab + capecitabine	1 time	976.6843	519.1815	1954.2796	358.7745	Gamma	Calculated
Cost of Computed tomography (CT) scan	Every 9 weeks	142.8571	107.1429	178.57143	17.85714	Gamma	25% +/− rule
Laboratory tests							
Cost of blood work	Every 3 weeks	6.27	4.7025	7.8375	0.78375	Gamma	25% +/− rule
Cost of echocardiogram	Every 13 weeks	119.05	89.2875	148.8125	14.88125	Gamma	25% +/− rule
Cost of palliative care/End of life	1 time	7185.721	3592.86	10778.581	1796.43	Gamma	50% +/− rule
Utilities							
Progression-free under treatment		0.785746	0.484478	0.9346889	0.112553	Beta	Calculated
Treatment response		0.061	0.025215	0.0742449	0.012257	Beta	Calculated
Disease progression under treatment		0.538	0.195937	0.8475389	0.162901	Beta	Calculated
Disutilities							
Disease progression		−0.248	−0.28854	−0.0871499	−0.050348	Uniform	Calculated
Adverse events for pertuzumab + trastuzumab + docetaxel		−0.05553	−0.09841	−0.0156854	−0.02068	Uniform	Calculated
Adverse events for trastuzumab + docetaxel		−0.03953	−0.05795	−0.0112224	−0.011682	Uniform	Calculated
Adverse events for TDM1		−0.00851	−0.01246	−0.0024128	−0.002512	Uniform	Calculated
Adverse events for lapatinib + capecitabine		−0.01826	−0.03193	−0.00395	−0.006996	Uniform	Calculated
Adverse events for trastuzumab + lapatinib		−0.01716	−0.02629	−0.00425	−0.005509	Uniform	Calculated
Adverse events for trastuzumab + capecitabine		−0.04017	−0.0747	−0.0092301	−0.016367	Negative Beta/Uniform	Calculated
Shape and scale parameters							
OS shape (Gamma)							
OS shape for pertuzumab + trastuzumab + Docetaxel		0.543696	0.460437	0.6420112	0.09264	Gamma	Regression
OS shape for trastuzumab + docetaxel		0.576501	0.501151	0.6631802	0.082668	Gamma	Regression
OS shape for TDM1		0.474333	0.414168	0.5432391	0.065853	Gamma	Regression
OS shape for lapatinib + capecitabine		0.464784	0.356733	0.605561	0.126953	Gamma	Regression
OS shape for trastuzumab + lapatinib		0.588267	0.467906	0.7395881	0.138613	Gamma	Regression
OS shape for trastuzumab + capecitabine		0.451302	0.33817	0.6022825	0.134751	Gamma	Regression
OS scale (Lambda)							
OS scale for pertuzumab + trastuzumab + docetaxel		0.019241	0.016447	0.0225107	0.003094	Gamma	Regression
OS scale for trastuzumab + docetaxel		0.024996	0.021942	0.028476	0.003334	Gamma	Regression
OS scale for TDM1		0.032557	0.029043	0.0364946	0.003802	Gamma	Regression
OS scale for lapatinib + capecitabine		0.015981	0.0126	0.0202686	0.003912	Gamma	Regression
OS scale for trastuzumab + lapatinib		0.018985	0.015258	0.0236232	0.004268	Gamma	Regression
OS scale for trastuzumab + capecitabine		0.040966	0.032984	0.0508812	0.009131	Gamma	Regression
PFS shape (Gamma)							
PFS shape for pertuzumab + trastuzumab + docetaxel		0.621872	0.560543	0.6899112	0.066004	Gamma	Regression
OS shape (Gamma)							
OS shape for pertuzumab + trastuzumab + Docetaxel		0.543696	0.460437	0.6420112	0.09264	Gamma	Regression
OS shape for trastuzumab + docetaxel		0.576501	0.501151	0.6631802	0.082668	Gamma	Regression
OS shape for TDM1		0.474333	0.414168	0.5432391	0.065853	Gamma	Regression
OS shape for lapatinib + capecitabine		0.464784	0.356733	0.605561	0.126953	Gamma	Regression
OS shape for trastuzumab + lapatinib		0.588267	0.467906	0.7395881	0.138613	Gamma	Regression
OS shape for trastuzumab + capecitabine		0.451302	0.33817	0.6022825	0.134751	Gamma	Regression
OS scale (Lambda)							
OS scale for pertuzumab + trastuzumab + docetaxel		0.019241	0.016447	0.0225107	0.003094	Gamma	Regression
OS scale for trastuzumab + docetaxel		0.024996	0.021942	0.028476	0.003334	Gamma	Regression
OS scale for TDM1		0.032557	0.029043	0.0364946	0.003802	Gamma	Regression
OS scale for lapatinib + capecitabine		0.015981	0.0126	0.0202686	0.003912	Gamma	Regression
OS scale for trastuzumab + lapatinib		0.018985	0.015258	0.0236232	0.004268	Gamma	Regression
OS scale for trastuzumab + capecitabine		0.040966	0.032984	0.0508812	0.009131	Gamma	Regression
PFS shape for trastuzumab + docetaxel		0.555381	0.504406	0.6115074	0.054643	Gamma	Regression
PFS shape for TDM1		0.610611	0.55205	0.6753828	0.062925	Gamma	Regression
PFS shape for lapatinib + capecitabine		0.516163	0.422877	0.6300273	0.105689	Gamma	Regression
PFS shape for trastuzumab + Lapatinib		0.553842	0.480821	0.637952	0.080169	Gamma	Regression
PFS shape for trastuzumab + capecitabine		0.508397	0.410262	0.6300055	0.112114	Gamma	Regression
PFS scale (Lambda)							
PFS scale for pertuzumab + trastuzumab + docetaxel		0.052051	0.046527	0.0582304	0.005971	Gamma	Regression
PFS scale for trastuzumab + docetaxel		0.074128	0.067234	0.081729	0.007396	Gamma	Regression
PFS scale for TDM1		0.104256	0.093296	0.116504	0.011841	Gamma	Regression
PFS scale for lapatinib + capecitabine		0.03389	0.027966	0.0410695	0.006685	Gamma	Regression
PFS scale for trastuzumab + lapatinib		0.082315	0.069929	0.0968932	0.013757	Gamma	Regression
PFS scale for trastuzumab + capecitabine		0.115186	0.093703	0.1415951	0.024435	Gamma	Regression
Weekly probability of developing adverse events							
Pertuzumab + trastuzumab + docetaxel		0.002564	0.001282	0.0038466	0.000641	Beta	Calculated
Trastuzumab + docetaxel		0.001969	0.000984	0.0029528	0.000492	Beta	Calculated
TDM1		0.004039	0.00202	0.0060591	0.00101	Beta	Calculated
Lapatinib + capecitabine		0.0105	0.00525	0.0157506	0.002625	Beta	Calculated
Trastuzumab + Lapatinib		0.001941	0.000971	0.0029122	0.000485	Beta	Calculated
Trastuzumab + capecitabine		0.005766	0.002883	0.0086493	0.001442	Beta	Calculated
Discount rate – weekly		0.000662	0	0.000939	–	Uniform	Calculated

**Table 6:** Model input parameters for no drug wastage scenario.

Parameters	Unit	Baseline	Deterministic SA		Probabilistic SA		Assumptions
			Low	High	PSA (SD)	Distribution	
Medical visit							
Physician fees	Every 3 weeks	8.15	6.11	10.19	1.01875	Gamma	25% +/− rule
Acquisition cost of treatments							
Loading dose pertuzumab (840 mg)	1st week of treatment only	4564.42	2282.21	6846.63	1141.105	Gamma	50% +/− rule
Maintenance dose pertuzumab (420 mg)	Every 3 weeks starting from week 4	2282.21	1141.11	3423.32	570.5525	Gamma	50% +/− rule
Loading dose trastuzumab (8mg/kg) -	1st week of treatment only	1955.79	977.89	2933.68	488.9465	Gamma	50% +/− rule
Maintenance dose trastuzumab (6 mg/kg)	Every 3 weeks starting from week 4	1466.84	733.42	2200.26	366.7099	Gamma	50% +/− rule
Docetaxel (1 mg)	Every 3 weeks for 6 cycles	760.88	380.44	1141.33	190.2211	Gamma	50% +/− rule
Pegfilgrastim (6 mg)	Every 3 weeks for 6 cycles	662.68	331.34	994.02	165.67	Gamma	50% +/− rule
TDM1	Every 3 weeks	3754.05	1877.02	5631.07	938.5124	Gamma	50% +/− rule
Capecitabine (500 mg)	Assuming 2 weeks of treatment per cycle + 1 week rest	158.27	79.14	237.41	39.5675	Gamma	50% +/− rule
Lapatinib (1500 mg daily)	Weekly	684.18	342.09	1026.27	171.045	Gamma	50% +/− rule
Cost of the management of Adverse Events (grade 3/4)							
Pertuzumab + trastuzumab + docetaxel	1 time	6628.784	3351.325	15468.774	3029.362	Gamma	Calculated
Trastuzumab + docetaxel	1 time	4749.982	2411.924	11520.075	2277.038	Gamma	Calculated
T-DM1	1 time	2347.695	1166.942	4626.3412	864.8498	Gamma	Calculated
Lapatinib + capecitabine	1 time	601.4326	297.29	1160.6912	215.8503	Gamma	Calculated
Trastuzumab + lapatinib	1 time	321.70	158.9764	619.84961	115.2183	Gamma	Calculated
Trastuzumab + capecitabine	1 time	976.6843	519.1815	1954.2796	358.7745	Gamma	Calculated
Cost of Computed tomography (CT) scan	Every 9 weeks	142.8571	107.1429	178.57143	17.85714	Gamma	25% +/− rule
Laboratory tests							
Cost of blood work	Every 3 weeks	6.27	4.7025	7.8375	0.78375	Gamma	25% +/− rule
Cost of echocardiogram	Every 13 weeks	119.05	89.2875	148.8125	14.88125	Gamma	25% +/− rule
Cost of palliative care/End of life	1 time	7185.721	3592.86	10778.581	1796.43	Gamma	50% +/− rule
Utilities							
Progression-free under treatment		0.785746	0.484478	0.9346889	0.112553	Beta	Calculated
Treatment response		0.061	0.025215	0.0742449	0.012257	Beta	Calculated
Disease progression under treatment		0.538	0.195937	0.8475389	0.162901	Beta	Calculated
Disutilities							
Disease progression		−0.248	−0.28854	−0.0871499	−0.050348	Uniform	Calculated
Adverse events for pertuzumab + trastuzumab + docetaxel		−0.05553	−0.09841	−0.0156854	−0.02068	Uniform	Calculated
Adverse events for trastuzumab + docetaxel		−0.03953	−0.05795	−0.0112224	−0.011682	Uniform	Calculated
Adverse events for TDM1		−0.00851	−0.01246	−0.0024128	−0.002512	Uniform	Calculated
Adverse events for lapatinib + capecitabine		−0.01826	−0.03193	−0.00395	−0.006996	Uniform	Calculated
Adverse events for trastuzumab + lapatinib		−0.01716	−0.02629	−0.00425	−0.005509	Uniform	Calculated
Adverse events for trastuzumab + capecitabine		−0.04017	−0.0747	−0.0092301	−0.016367	Negative Beta/Uniform	Calculated
Shape and scale parameters							
OS shape (Gamma)							
OS shape for pertuzumab + trastuzumab + Docetaxel		0.543696	0.460437	0.6420112	0.09264	Gamma	Regression
OS shape for trastuzumab + docetaxel		0.576501	0.501151	0.6631802	0.082668	Gamma	Regression
OS shape for TDM1		0.474333	0.414168	0.5432391	0.065853	Gamma	Regression
OS shape for lapatinib + capecitabine		0.464784	0.356733	0.605561	0.126953	Gamma	Regression
OS shape for trastuzumab + lapatinib		0.588267	0.467906	0.7395881	0.138613	Gamma	Regression
OS shape for trastuzumab + capecitabine		0.451302	0.33817	0.6022825	0.134751	Gamma	Regression
OS scale (Lambda)							
OS scale for pertuzumab + trastuzumab + docetaxel		0.019241	0.016447	0.0225107	0.003094	Gamma	Regression
OS scale for trastuzumab + docetaxel		0.024996	0.021942	0.028476	0.003334	Gamma	Regression
OS scale for TDM1		0.032557	0.029043	0.0364946	0.003802	Gamma	Regression
OS scale for lapatinib + capecitabine		0.015981	0.0126	0.0202686	0.003912	Gamma	Regression
OS scale for trastuzumab + lapatinib		0.018985	0.015258	0.0236232	0.004268	Gamma	Regression
OS scale for trastuzumab + capecitabine		0.040966	0.032984	0.0508812	0.009131	Gamma	Regression
PFS shape (Gamma)rowhead							
PFS shape for pertuzumab + trastuzumab + docetaxel		0.621872	0.560543	0.6899112	0.066004	Gamma	Regression
PFS shape for trastuzumab + docetaxel		0.555381	0.504406	0.6115074	0.054643	Gamma	Regression
PFS shape for TDM1		0.610611	0.55205	0.6753828	0.062925	Gamma	Regression
PFS shape for lapatinib + capecitabine		0.516163	0.422877	0.6300273	0.105689	Gamma	Regression
PFS shape for trastuzumab + Lapatinib		0.553842	0.480821	0.637952	0.080169	Gamma	Regression
PFS shape for trastuzumab + capecitabine		0.508397	0.410262	0.6300055	0.112114	Gamma	Regression
PFS scale (Lambda)							
PFS scale for pertuzumab + trastuzumab + docetaxel		0.052051	0.046527	0.0582304	0.005971	Gamma	Regression
PFS scale for trastuzumab + docetaxel		0.074128	0.067234	0.081729	0.007396	Gamma	Regression
PFS scale for TDM1		0.104256	0.093296	0.116504	0.011841	Gamma	Regression
PFS scale for lapatinib + capecitabine		0.03389	0.027966	0.0410695	0.006685	Gamma	Regression
PFS scale for trastuzumab + lapatinib		0.082315	0.069929	0.0968932	0.013757	Gamma	Regression
PFS scale for trastuzumab + capecitabine		0.115186	0.093703	0.1415951	0.024435	Gamma	Regression
Weekly probability of developing adverse events							
Pertuzumab + trastuzumab + docetaxel		0.002564	0.001282	0.0038466	0.000641	Beta	Calculated
Trastuzumab + docetaxel		0.001969	0.000984	0.0029528	0.000492	Beta	Calculated
TDM1		0.004039	0.00202	0.0060591	0.00101	Beta	Calculated
Lapatinib + capecitabine		0.0105	0.00525	0.0157506	0.002625	Beta	Calculated
Trastuzumab + Lapatinib		0.001941	0.000971	0.0029122	0.000485	Beta	Calculated
Trastuzumab + capecitabine		0.005766	0.002883	0.0086493	0.001442	Beta	Calculated
Discount rate – weekly		0.000662	0	0.000939	–	Uniform	Calculated

## 2 Experimental design, materials, and methods

All costs presented were direct medical costs from the perspective of the Taiwanese National Health Insurance Administration (TNHIA). Cost for physician visits, treatments, CT scans, and laboratory tests were obtained from the website of TNHIA and personal communication. Most HER-2 targeted treatments are dosed based on weight or body surface area (BSA), so the treatment costs were then calculated based on the average height and weight for the Taiwanese female population. The average height and weight were obtained from the Ministry of Health and Welfare, Department of Statistics in Taiwan. Two cost strategies were developed—with drug wastage and without drug wastage. In the drug wastage scenario, the price per vial for each intravenous drug was not broken down. For example, the calculated dosage for docetaxel is 119.25 mg, the total costs for 1 vial of 80mg plus the cost for 2 vials of 20 mg which equals to 120 mg will represent the costs for 119.25.

mg docetaxel. However, in the no drug wastage scenario, the price per vial for each intravenous drug was broken down. For example, the calculated dosage for docetaxel is 119.25 mg and the costs for 1.49 vial of 80mg docetaxel will represent the costs for 119.25mg docetaxel. Cost of palliative care was obtained from Taiwanese National Health Insurance Research Database.

Transition probabilities for progression and mortality were obtained from published clinical trials. Individual patient data (IPD) were obtained from the PFS and OS Kaplan-Meier curves from these clinical trials using a method described previously. Five standard parametric distributions were fitted to the IPD: exponential, Weibull, Gompertz, lognormal, and log-logistic. The log-logistic model was then selected and used to reconstructed IPD and derived equations for transition probabilities using the shape and scale parameters of the fitted model because it had the greatest model fit which was evaluated based on AIC and BIC. Utilities were obtained from published literature and clinical trials.
